# Review of the Emotions and the Will

**Published:** 1860

**Authors:** Alexander Bain

**Affiliations:** Examiner in Logic and Moral Philosophy in the University of London


*Review of the Emotions and the Will.* By ALEXANDER BAIN, A. M., Examiner in Logic and Moral Philosophy in the University of London.—*London*, 1859. 8vo. pp. 646.

After the controversy between the Neptunists and the Vulcanists had been long carried on without definite results, there came a reaction against all speculative geology. Reasoning without adequate data, having led to nothing, inquirers went into the opposite extreme, and for a length of time, confining themselves wholly to collecting data, relinquished reasoning. The Geological Society of London was formed with the express object of accumulating evidence; for many years hypotheses were forbidden at its meetings; and only of late have attempts to organize the mass of observations into consistent theory been tolerated.

This reaction and subsequent re-reaction well illustrate the recent history of English thought in general. The time was when our countrymen occupied themselves, certainly to as great an extent as any other people, with all those high questions which present themselves to the human intellect; and, indeed, a glance at the various systems of philosophy that are or have been current on the Continent, suffices to show how much other nations owe to the speculation of our ancestors. For a generation or two, however, these more abstract subjects have fallen into neglect, and, among those who plume themselves on being "practical," even into contempt. Partly, perhaps, a natural accompaniment of our rapid material growth, this intellectual phase has been in great measure due to the exhaustion of argument, and the necessity for better data. Not so much with a conscious recognition of the end to be subserved as from an unconscious subordination to that rhythm traceable in social changes as in other things, an era of theorizing without observing, has been followed by an era of observing without theorizing. During this long-continued devotion to concrete science, an immense quantity of raw material for abstract science has been accumulated; and now there is obviously commencing a period in which this accumulated raw material will be organized into consistent theory. On all sides—equally in the inorganic sciences, in the science of life, and in the science of society—may we note the tendency to pass from the superficial and empirical to the more profound and rational.

In Psychology this change is conspicuous. The facts brought to light by anatomists and physiologists during the last fifty years are at length being used towards the interpetration of this highest class of biological phenomena; and already there is promise of a great advance. The work of Mr. Alexander Bain, of which the second volume has been recently issued, may be regarded as especially characteristic of the transition. It gives us in orderly arrangement the great mass of evidence supplied by modern science towards the building up of a coherent system of mental philosophy. It is not in itself a system of mental philosophy, properly so called; but a classified collection of materials for such a system, presented with that method and insight which scientific discipline generates, and accompanied with occasional passages of an analytical character. It is indeed that which it in the main professes to be—a natural history of the mind. Were we to say that the researches of the naturalist who collects and dissects and describes species, bear the same relation to the researches of the comparative anatomist tracing out the laws of organization, which Mr. Bain's labours bear to the labours of the abstract phsychologist, we should be going somewhat too far; for Mr. Bain's work is not wholly descriptive.—Still, however, such an analogy conveys the best general conception of what he has done; and serves most clearly to indicate its needfulness. For as, before there can be made anything like true generalizations respecting the classification of organisms and the laws of organization, there must be an extensive accumulation of the facts presented in numerous organic bodies; so, without a tolerably complete delineation of mental phenomena of all orders, there can scarcely arise any adequate theory of the mind. Until recentiy, mental science has been pursued much as physical science was pursued by the ancients: not by drawing conclusions from observation and experiment, but by drawing them from certain arbitrary *a priori* assumptions. This course, long since abandoned in the one case with immense advantage, is gradually being abandoned in the other; and the treatment of Psychology as a division of natural history, shows that the abandonment will soon be complete.

Estimated as a means to higher results, Mr. Bain's work is of great value. Of its kind it is the most scientific in conception, the most catholic in spirit, and the most complete in excution.—Not only does it delineate the various classes of mental phenomena as seen under that stronger light thrown on them by modern science; but it includes in the picture much which previous writers had omitted—partly from prejudice, partly from ignorance. We refer more especially to the participation of the various bodily organs in the mental changes, and the addition to these mental changes of those many secondary ones which the action of the bodily organs generate. Mr. Bain has, we believe, been the first adequately to appreciate the importance of this element in our states of consciousness; and it is one of his chief merits that he shows how constant and large an element it is.—Further, the relations of voluntary and involuntary action are elucidated in a way that was not possible to writers unacquainted with the modern doctrine of reflex action. Add to which, that some of the analytical passages that here and there occur contain important ideas.

Valuable, however, as is Mr. Bain's work, we regard it as essentially transitional. It fulfils the office of presenting in a digested form the results of a period of observation; adds to these results much well-delineated fact collected together by himself; arranges new and old materials with that more scientific method which the discipline of our times has fostered; and so prepares the way for better generalizations. But almost of necessity its classifications and conclusions are provisional. In the growth of each science, we may observe, that not only is correct observation needful for the formation of true theory; but that true theory is needful as a preliminary to correct observation. Of course we do not intend this assertion to be taken literally; but as a strong expression of the fact that the two must advance hand in hand. The first crude theory or rough classification, based on very limited knowledge of the phenomena, is requisite as a means of reducing the phenomena to some kind of order; and as supplying a conception with which fresh phenomena may be compared, and their agreement or disagreement noted. Incongruities being by-and-bye made manifest by wider examination of cases, there eventually comes such modification of the theory as brings it into a nearer correspondence with the evidence. This reacts to the further advance of observation. More extensive and complete observation brings additional corrections of theory. And so on till the truth is reached. In mental science the systematic and extensive collection of facts having but recently commenced, it is not to be expected that the results can be at once rightly formulated. All that may be looked for are approximate generalizations which will presently serve for the better directing of inquiry. And hence, even were it not now possible to say in what way it does so, we might be tolerably certain that the work of Mr. Bain bears the stamp of the inchoate state of Psychology.

We think, however, that it will not be difficult to find in what respects its organization is provisional; and at the same time to show what must be the nature of a more complete organization. We propose here to attempt this: illustrating our positions from his recently issued second volume.

Is it possible to make a true classification without the aid of analysis? or must not there be an analytical basis to every true classification? Can the right relations of things be determined by reference to their more obvious characteristics? or does it not commonly happen that certain hidden characteristics, on which the more obvious ones depend, are the truly significant ones?— This is the preliminary question which a glance at Mr. Bain's scheme of the emotions suggests.

Though not avowedly, yet by implication, Mr. Bain assumes that a true conception of the nature, the order and the mutual relations of the emotions, may be arrived at by contemplating their conspicuous objective and subjective characters, as displayed in the adult. After pointing out that we lack those means of classification which serve in the case of the sensations, he says—

"In these circumstances we must turn our attention to the *manner of diffusion* of the different passions and emotions, in order to obtain a basis of classification analogous to the arrangement of the sensations. If what we have already advanced on that subject be at all well founded, this is the genuine turning point of the method to be chosen, for the same mode of diffusion will always be accompanied by the same mental experience, and each of the two aspects would identify, and would be evidence of the other. There is, therefore, nothing so thoroughly characteristic of any state of feeling as the nature of the diffusive wave that embodies it, or the various organs specially roused into action by it, together with the manner of the action. The only drawback is our comparative ignorance, and our inability to discern the precise character of the diffusive currents in every case; a radical imperfection in the science of mind as constituted at present.

"Our own consciousness, formerly reckoned the only medium of knowledge to the mental philosopher, must, therefore, be still referred to as a principal means of discriminating the varieties of human feeling. We have the power of noting agreement and difference among our conscious states, and on this we can raise a structure of classification. We recognize such generalities as pleasure, pain, love, anger, through the property of mental or intellectual discrimination that accompanies in our mind the fact of an emotion. A certain degree of precision is attainable by this mode of mental comparison and analysis; the farther we can carry such precision the better; but that is no reason why it should stand alone to the neglect of the corporeal embodiments through which one mind reveals itself to others. The companionship of inward feeling with bodily manifestation, is a fact of the human constitution, and deserves to be studied as such; and it would be difficult to find a place more appropriate than a treatise on the mind, for setting forth the conjunctions and sequences traceable in this department of nature. I shall make no scruple in conjoining with the description of the mental phenomena, the physical appearances, in so far as I am able to ascertain them.

"There is still one other quarter to be referred to in settling a complete arrangement of the emotions, namely, the varieties of human conduct, and the machinery created in subservience to our common susceptibilities. For example, the vast superstructure of fine art has its foundations in human feeling, and in rendering an account of this we are led to recognize the interesting group of artistic or æsthetic emotions. The same outward reference to conducts and creations brings to light the so-called moral sense in man, whose foundations in the mental system have accordingly to be examined.

"Combining together these various indications, or sources of discrimination—outward objects, diffusive mode or expression, inward consciousness, resulting conduct and institutions—I

adopt the following arrangement of the families or natural orders of emotion."

Here, then, we have confessedly adopted, as bases of classification, the most manifest characters of the emotions as discerned subjectively and objectively. The mode of diffusion of an emotion is one of its outside aspects; the institutions it generates form another of its outside aspects; and though the peculiarities of the emotion as a state of consciousness, may seem to substitute its intrinsic and ultimate nature, yet such peculiarities as are perceptible in an ordinary act of self-consciousness, must also be classed as in a sense superficial peculiarities. It is a familiar fact that various intellectual states of consciousness turn out, when analyzed, to have a nature widely unlike that which at first appears; and we believe the like will prove true of emotional states of consciousness. Just as one concept of space, which seems on first consideration to be a definite, undecomposable concept, is yet resolvable into experiences quite different from that resulting state of consciousness which we call space; so, in all probability, the sentiment of affection or reverence is compounded of elements that are severally distinct from the whole which they make up. And much as a classification of our ideas which dealt with the idea of spacc as though it were ultimate, would be a classification of ideas by their externals; so is a classification of our emotions, which, regarding them as simple, describes their aspect in ordinary consciousness, a classification of emotions by their externals.

Thus, then, Mr. Bain's grouping is throughout determined by the most manifest attributes—those objectively displayed to us in the natural language of the emotions, and in the social phenomena that results from them, and those subjectively displayed in the aspects they assume in an unanalytical consciousness. And the question is—Can they be correctly grouped after this method?

We think not; and we believe that had Mr. Bain carried farther an idea with which he has set out, he would have seen that they cannot. As already said, he avowedly adopts "the natural history method;" not only referring to it in his preface, but in his first chapter giving examples of botanical and zoological classifications, as illustrating the mode in which he proposes to deal with the emotions. This we conceive to be quite a philosophical conception; and have only to regret that Mr. Bain has overlooked some of its most important implications. For in what has essentially consisted the progress of natural history classification? In the abandonment of grouping by external conspicuous characters; and in the making certain internal, but ill-essential characters, the bases of groups. Whales are not now arranged along with fish, because in their general forms and habits they resemble fish; but they are arranged with mammals, because the type of their organization, as ascertained by dissection, corresponds with that of mammals. No longer considered as sea-weeds in virtue of their forms and modes of growth, zoophytes are now shown, by examination of their economy, to belong to the animal kingdom. It is found, then, that true classification involves analysis. It has turned out that the earlier classifications, guided by general resemblances, though containing a large amount of truth, and though very useful provisionally, were yet in many cases radically wrong; and that the real affinities of organisms, and the real homologies of their parts, were to be made out only by examining their hidden structures. Another fact of great significance in the history of classification is also to be noted:—we mean the fact that very frequently the true relations of organisms cannot be made out even by an exhaustive analysis, if that analysis is confined to the adult structure; but that in many cases it is needful to examine the structure in its earlier stages, and even in its embryotic stage. So difficult was it, for instance, to determine the true position of the Cirrhipedia among animals, so long as mature individuals only were examined, that Cuvier fell into the mistake of classing them with Mollusca, even after an anatomical examination of them; and it was only when their early forms were discovered, that they were clearly proved to belong to the Crustacea. So important, is the study of development as a means to classification, that the first zoologists now hold it to be the only absolute criterion.

Here, then, in the advance of natural history classification, are two fundamental facts, which should be borne in mind in classifying the emotions. If, as Mr. Bain rightly assumes, the emotions are to be grouped after the natural history method, then should it be the natural history method in its complete, and not in its rude form. Mr. Bain will doubtless agree in the position, that a correct account of the emotions in their natures and relations, must correspond with a correct account of the nervous system—must form another side of the same ultimate facts.—Structure and function must necessarily harmonize. Structures which have with each other certain ultimate connexions, must have functions that have answering connexions. Structures that have arisen in certain ways, must have functions that have arisen in parallel ways. And hence if analysis and development are needful for the right interpetration of structures, so must they be needful for the right interpretation of functions. Just as a scientific description of the digestive organs must include not only their obvious forms and connexions, but their microscopic characters, and also the ways in which they severally result by differentiation from the primitive mucous membrane; so must a scientific account of the nervous system include its general arrangements, its minute structure, and its mode of evolution ; and so must a scientific account of nervous actions include the answering three elements. Alike in classing separate organisms, and in classing the parts of the same organism, the complete natural history method involves ultimate analysis, aided by development; and Mr. Bain, in not basing his classification of the emotions upon characters reached through these aids, has fallen much short of the conception with which he set out.

"But," it will perhaps be asked, "how are the emotions to be analysed, and their modes of evolution to be ascertained ? Different animals, and different organs of the same animal, may be readily compared in their internal and microscopic structures, as also in their development ; but functions, and especially such functions as the emotions, do not admit of such comparisons."

It must be admitted that the application of these methods is in this case by no means so easy. Though we can note differences and similarities in the internal formations of two animals, it is difficult to contrast with any precision their mental states.—Though the true morphological relations of organs may be made out by the observation of embryos, yet, where those organs are inactive before birth, we cannot completely trace the history of their actions. Further, it is obvious that the pursuance of inquiries of the kind indicated, raises questions which science is not yet prepared to answer; as, for instance—Whether all nervous functions, in common with all other functions, do not arise by gradual differentiations, as their organs do? Whether the emotions are not, therefore, to be regarded as divergent modes of action that have become unlike by successive modifications? Whether, as two organs which have originally budded out of the same membrane have not only become different as they developed, but have also severally become highly compound internally, though externally simple; so two emotions, simple and near akin in their roots, may not only have grown unlike, but may also have grown involved in nature, though seeming homogeneous to consciousness. And here, indeed, in the inability of existing science to answer these questions which underlie a true psychological classification, we see most clearly how purely provisional any present classification is likely to be.

Nevertheless, even now, classification may be aided by development and ultimate analysis to a considerable extent; and the great defect in Mr. Bain's work is, that he has not systematically availed himself of them as far as possible. Thus we may, in the first place, study the evolutions of the emotions up through the various grades of the animal kingdom; observing which of them are earliest and exist with the lowest organization and intelligence; in what order the others accompany higher endowments, and how they are severally related to the conditions of life. In the second place, we may note the emotional differences between the lower and the higher human races—may regard as earlier and simpler those feelings which are common to both, and as later and more compound those which are characteristic of the most civilized. In the third place, we may observe the order in which the emotions unfold during the progress from infancy to maturity. And lastly, comparing together these three kinds of emotional development, displayed in the ascending grades of the animal kingdom, in the advance of the civilized races, and in individual growth, we may see in what respects they harmonize, and what are the implied general truths.

Having gathered together and generalized these several classes of facts, we should have better means of studying the emotions analytically. Setting out with the unquestionable assumption that every new form of emotion making its appearance in the individual or the race, is a modification of some pre-existing emotion, or a compounding of several pre-existing emotions, we should be greatly aided by knowing what always are the pre-existing emotions. When, for example, we find that the love of accumulation is absent in the lower animals and in infancy—when we see that an infant may exhibit anger, fear, wonder, while yet it manifests no desire of permanent possession, and that without any conception of property a brute may show attachment, jealousy, love of approbation; we may suspect that the love of property is compounded out of simpler and deeper feelings. We may conclude that as, when a dog hides a bone, there must exist in him a prospective gratification of hunger, so there must similarly at first, in all cases where anything is secured or taken possession of, exist an ideal excitement of the feeling which that thing will gratify. We may further conclude that when the intelligence is such that a variety of objects come to be utilized for different purposes—when, as among savages, divers wants are satisfied through the articles appropriated for weapons, shelter, clothing, ornament; the act of appropriating comes to be one constantly involving agreeable associations, and one which is, therefore, pleasurable, irrespective of the end subserved. Add to which, that when, as in civilized life, the property acquired is of a kind not conducing to one order of gratification in particular, but is capable of administering to all gratifications, the pleasure of acquiring property grows more distinct from the various pleasures subserved—more completely differentiated into a separate emotion.

The illustration, roughly as it is sketched, will show what we mean by the use of comparative psychology in aid of classification. Ascertaining by induction the actual order of evolution ot the emotions, we are led to suspect this to be their order of successive dependence; and are so led to recognize their order of ascending complexity, and by consequence their true groupings.

And thus, in the very process of arranging the emotions into grades, beginning with those involved in the lowest forms of conscious activity and ending with those peculiar to the adult civilized man, the way is opened to that ultimate analysis which alone can lead us to the true science of the matter. For when we find not only that there exist in man various feelings which do not exist in a child, but also that the European is characterized by some sentiments which are wholly or in great part absent from the savage—when we see that, besides the new emotions that arise spontaneously as the individual becomes completely organized, there are new emotions making their appearance in the more advanced divisions of our race—we are led to ask—How are new emotions generated? It is notorious that some of the lowest savages, as the Australians, have not even the ideas of justice or mercy. They have neither words for them nor can they be made to conceive them; and the manifestation of them by Europeans they ascribe to fear or cunning. Further, it is a familiar fact that there are æsthetic emotions, common among ourselves, that are scarcely in any degree experienced by the inferior races, as, for instance, those produced by music. To which instances may be added the less marked, but more numerous contrasts that exist between the various civilized nations in the degrees of their several emotions. And if it is manifest, not only that all the emotions are capable of being permanently modified in the course of successive generations, but also that what must be classed as new emotions may be brought into existence; then it is clear that nothing like a true conception of the emotions is to be obtained, until we understand how they are evolved.

Comparative psychology, while it raises this inquiry, also prepares the way for answering it. For when observing the differences between races we can scarcely at the same time fail to observe how these differences correspond with differences in their conditions of existence, and therefore in their daily experienees. To take first the minor illustrations, we see how conquest fosters the martial feeling, and how a love of the sea is generated by conditions that necessitate maritime activity. We see how nations that have long had free institutions acquire an independence of character strongly contrasted with that servility characterizing nations that have always been governed despotically. But chiefly in the differences between the emotional natures of savage and civilized, may we note the effects of changed conditions. Among the lowest races of men, the love of property stimulates to the attainment only of such things as satisfy imme diate desires or desires of immediate future. Improvidence is the rule: there is little effort to meet remote contingencies. But the growth of established societies having gradually given security of possession, there has been an increasing tendency to provide for coming years; a constant exercise of the feeling which is satisfied by a provision for the future; and a growth of this feeling so great that it now prompts accumulation to an extent beyond what is needful. Note, again, that under the discipline of social life—under a comparative abstinence from aggressive actions, and a performance of those mutually serviceable actions implied by the division of labour—there has been a development of those gentle emotions of which inferior races exhibit but the rudiments. Savages delight rather in giving pain than pleasure—are almost devoid of sympathy. While among ourselves philanthropy organizes itself in laws, establishes numerous institutions, and dictates countless private benefactions.

From which and other like facts, does it not seem an unavoidable inference that new emotions are developed by new experiences—new habits of life? All are familiar with the truth, that in the individual each feeling may be strengthened by performing those actions which it prompts; and to say that the feeling is *strengthened*, is to say that it is in part *made* by these actions. We know further, that not unfrequently, individuals, by persistence in certain special courses of conduct, acquire special liking for such courses, disagreeable as these may be to others; and these whims, hobbies, or morbid tastes, are nothing but incipient emotions corresponding to these special activities. We know that emotional characteristics, in common with all others, are more or less clearly hereditary; and that the differences between civilized nations descended from the same stock, show us the cumulative results of small modifications hereditarily transmitted. And when we see that between savage and civilized races which diverged from each other in the remote past, and have for a hundred generations followed modes of life becoming ever more unlike, there exist still greater emotional contrasts; is it not clear that the more or less distinct emotions which characterize the civilized races, are the organized results of certain daily-repeated combinations of mental states which social live involves? Must we not say that habits not only modify emotions in the individual, and not only beget tendencies to like habits and accompanying emotions in descendants, but that when the conditions of the race make the habits persistent, this progressive modification may go on to the extent of producing emotions so far distinct as to seem new? And if so, we may suspect that such new emotions, and by implication all emotions analytically considered, consist of aggregated and consolidated groups of those simpler feelings which habitually occur together in experience: that not only do they result from combined experiences, but are constituted of them. When in the circumstances of any race some one kind of action or set of actions, sensation or set of sensations, is usually followed or accompanied by various other sets of actions or sensations, and so entails a large mass of pleasurable or painful states of consciousness; these by frequent repetition become so connected together that the initial action or sensation brings the ideas of all the rest crowding into consciousness, producing in a degree the pleasure or pains that have been felt in reality. And when this relation is not unfrequently repeated in the individual, but occurs in successive generations, all the many nervous actions involved tend to grow organically connected. They become incipiently reflex; and on the occurrence of the appropriate stimulus, the whole nervous apparatus which in past generations was brought into activity by this stimulus, becomes nascently excited. Even while yet there have been no individual experiences, a vague feeling of pleasure or pain is produced, constituting what we may call the body of the emotion. And when the experiences of past generations come to be repeated in the individual, the emotion gains both strength and definiteness; and is accompanied by the appropriate specific ideas.

This view of the matter, which we believe the established truths of Physiology and Psychology unite in indicating, and which is the view that generalizes the phenomena of habit, of national characteristics, of civilization in its moral aspects, at the same time that it gives us a conception of emotion in its origin and ultimate nature, is well illustrated in the mental modifications undergone by animals. It is, for instance, a well known fact, that on newly discovered lands not inhabited by man, birds are so devoid of fear as to allow themselves to be knocked over with sticks; but that in the course of generations they acquire such a dread of man as to fly on his approach; and that this dread is manifested by young as well as old. Now, in the first place, it is clear that this change is the result of accumulated experiences; and that each experience has a share in producing it. Whence it follows that in each individual bird that had escaped with injuries inflicted by man, or that had been alarmed by the outcrys of other members of the flock (gregarious creatures of any intelligence being necessarily more or less sympathetic), there had been established an association of ideas between the human aspect and the pains, direct and indirect, suffered from human agency. It follows further, that the state of consciousness which impels the bird to take flight, is at first nothing more than an ideal reproduction of those painful impressions which before followed man's approach; that such ideal reproduction has become more vivid and more massive as the quantity of painful experiences has increased; and that thus the emotion in its incipient state is nothing else than an aggregation of the revived pains before experienced. In the second place remark, that as in the course of generations the young birds of this race begin to display a fear of man before yet they have been injured by him, it is an unavoidable inference that the nervous system of the race has been organically modified by these experiences: we have no choice but to conclude that when a young bird is thus led to fly, it is because the impression produced on its senses by the approaching man, entails through an incipiently reflex action, a partial excitement of all those nerves which in its ancestors had been excited under the like conditions; that this partial excitement has its accompanying painful consciousness; and that the vague, painful consciousness, thus arising, constitutes emotion proper—*emotion undecomposable into specific experiences, and therefore seemingly homogeneous.*

If such be the true explanation of the fact in this case, then is it in all cases. If emotion is so generated here, then is it so generated throughout. We must perforce conclude, that the emotional modifications displayed by different nations, and those higher emotions by which civilized are distinguished from savage, are to be accounted for on the same principle. And concluding this, we are led strongly to suspect that the emotions in general have severally a like origin.

Perhaps we have now made sufficiently clear what we mean by the study of the emotions through analysis and development.

At the same time we venture to think we have justified the positions that, without analysis, aided by development, there cannot be a true natural history of the emotions; and that a natural history of the emotions based on external characters can be but provisional. Mr. Bain, in confining himself to an account of the emotions as they exist in the adult civilized man, has neglected those classes of facts out of which the science of the matter must chiefly be built. It is true that he has treated of habits as modifying emotions in the individual; but he has not recognized the fact, that where conditions render habits persistent in successive generations, such modifications are cumulative: he has not hinted that the modifications produced by habit are emotions in the making. It is true also, that he occasionally refers to the characteristics of children; but he does not systematically trace the changes through which childhood passes into manhood, as throwing any light on the order and genesis of the emotions.—It is further true that he here and there instances national traits in illustration of his subject; but these stand as isolated facts, having no general significance; there is no hint of any relation between them and the national circumstances; while all those many moral contrasts between lower and higher races which throw great light on classification, are overlooked. And once more, it is true that many passages of his work, and sometimes, indeed, whole sections of it, are analytical; but his analyses are incidental—they do not underlie his entire scheme, but are here and there added to it. In brief, he has written a Descriptive Psychology, which does not appeal to Comparative Psychology and Analytical Psychology for its leading ideas. And in doing this, he has not only of necessity omitted much that should be included in a natural history of the mind, but to that part of the subject with which he has dealt, he has given an organization that is necessarily superficial.

Even leaving out of view the absence of those methods and criteria on which we have been insisting, it appears to us that meritorious as is Mr. Bain's book in its details, it is seriously defective in some of its leading ideas. The first paragraphs of his first chapter quite startled us by the strangeness of their definitions—a strangeness which can scarcely be ascribed to laxity of expression. The paragraphs run thus:—

"Mind is comprised under three heads—Emotion, Volition, and Intellect.

"Emotion is the name here used to comprehend all that is understood by feelings, states of feeling, pleasures, pains, passions, sentiments, affections. Consciousness, and conscious states also, for the most part, denote modes of emotion, although there is such a thing as the Intellectual consciousness.

"Volition, on the other hand, indicates the great fact that our pleasures and pains, which are not the whole of our emotions, prompt to action, or stimulate the active machinery of the living frame-work to perform such operations as procure the first and abate the last. To withdraw from a scalding heat and cling to a gentle warmth, are exercises of volition."

To take first the last of these definitions, we cannot but feel astonished that Mr. Bain, familiar as he is with the phenomena of reflex action, should have so expressed himself as to include a great part of them along with the phenomena of volition. Not only does he seem to be ignoring the discriminations of modern science, and returning to the vague conceptions of the past, but he is comprehending under volition, what even the popular speech would hardly bring under it. If you were to blame any one for snatching his foot from the scalding water into which he had inadvertently put it, he would tell you that he could not help it; and his reply would be endorsed by the general experience that the withdrawal of a limb from contact with something extremely hot is quite involuntary—that not only does it take place without volition, but that it takes place in defiance of an effort of will to maintain the contact. How, then, can that be instanced as an example of volition, which occurs even when volition is strongly antagonistic? We are quite aware that it is impossible to draw any absolute line of demarcation between automatic actions and those which are not automatic. Doubtless we may pass gradually from the purely reflex through the consensual to the voluntary. Taking the case Mr. Bain cites, it is manifest that from a heat of such moderate degree that the withdrawa from it is wholly voluntary, we may advance by infinitesimal steps to a heat such as to compel involuntary withdrawal; and that there is a stage at which the voluntary and involuntary actions are mixed. But the difficulty of absolute discrimination is no reason for neglecting the broad general contrast, any more than it is for confounding light with darkness. If we are to include as examples of volition, all cases in which pleasures and pains "stimulate the active machinery of the living framework to perform such operations as procure the first and abate the last," then must we consider sneezing and coughing as examples of volition. If Mr. Bain does not mean this, then his mode of expressing himself is lax to a degree that is especially improper in a scientific work. If he does mean it, then his conception of will seems to us more vague even than that which is current.

A parallel criticism applies to his definition of Emotion. Here, too, he has departed from the ordinary acceptation of the word, and, as we think, in the wrong direction. Whatever may be the interpretation that is justified by its derivation, the word Emotion has come generally to mean that kind of feeling which is not a direct result of any action on the organism, but is either an indirect result of such action or arises quite apart from any such action. We commonly use it to indicate those sentient states which are independently generated in consciousness; as distinguished from those generated in our corporeal framework, and known as sensations. Now this distinction, tacitly made in common parlance, is one which Psychology cannot well reject; but one which it must adopt, and to which it must give scientific precision. But Mr. Bain appears to ignore any such distinction. Under the term "emotion" he includes not only passions, sentiments, affections, but all "feelings, states of feeling, pleasures, pains"—that is, all sensations. This does not appear to be a mere lapse of expression; for when in the opening sentence he asserts that "mind is comprised under the three heads—Emotion, Volition, and Intellect," he of necessity implies that sensation is included under one of these heads; and as it cannot be included under volition or intellect, it must be classed with emotion: as it clearly is in the next sentence.

We cannot but think this an entirely retrograde step. Though distinctions which have been established in popular thought and language, are not unfrequently merged in the higher generalizations of science (as, for instance, when crabs and worms are grouped together in the sub-kingdom *Annulosa*), yet science very generally recognizes the validity of these distinctions, as real though not fundamental. And so in the present case. Such community as is disclosed between sensation and emotion, by an ultimate analysis, must not shut out the broad contrast that exists between them. If there needs a wider word, as there does, to signify any sentient state whatever, then we may fitly adopt for this purpose the word currently so used, namely, "Feeling." And considering as Feelings all that great division of mental states which we do not class as Cognitions, we may then separate this great division into the two orders, Sensations and Emotions.

And here we may, before concluding, briefly indicate the leading outlines of a classification which reduces this distinction to a scientific form, and developes it somewhat further—a classification which, while suggested by certain fundamental traits reached without a very laborious inquiry, is yet, we believe, in harmony with that disclosed by detailed analysis.

Leaving out of view the Will, which is a simple homogeneous mental state, forming the link between feeling and action, and not admitting of subdivisions, our states of consciousness fall into the two great classes of Cognitions and Feelings.

Cognitions, which are those modes of mind in which we are occupied with the *relations* that subsist among our feelings, are divisible into three great sub-classes.

*Presentative cognitions;* or those in which consciousness is occupied in localizing a sensation impressed on the organism—occupied, that is, with the relation between this presented mental state and those other presented mental states which make up our consciousness of the part affected: as when we cut ourselves.

*Presentative-representative cognitions;* or those in which consciousness is occupied with the relation between a sensation or group of sensations and the representations of those various other sensations that accompany it in experience. This is what we commonly call perception—an act in which, along with certain impressions presented to consciousness, there arise in consciousness the ideas of certain other impressions that are ordinarily connected with it: as when from its visible form and colour we mentally endow an orange with all its other attributes.

*Representative cognitions;* or those in which consciousness is occupied with the relations among ideas or represented sensations: as in all acts of reflection.

FEELINGS, which are those modes of mind in which we are occupied, not with the relations subsisting between our sentient states, but with the sentient states themselves, are divisible into three parallel sub-classes.

*Presentative feelings*, ordinarily called sensations, are those mental states in which instead of regarding a corporeal impression as of this or that kind, or as located here or there, we contemplate it as pleasure or pain: as when eating.

*Presentative-representative feelings*, embracing a great part of what we commonly call emotions, are those in which a sensation or group of sensations arouses a vast aggregation of represented sensations, partly of individual experience, but chiefly deeper than individual experience, and, consequently, indefinite. The emotion of terror may serve as an example. Along with certain impressions made on the eyes or ears, or both, are recalled into consciousness a crowd of the pains to which such impressions have before been the antecedents; and when the relation between such impressions and such pains has been habitual in the race, the definite ideas of such pains which individual experience has given are accompanied by the indefinite pains that result from inherited experience—vague feelings which we may call organic representations. In the infant, crying at a strange sight or sound while yet in the nurse's arms, we see these organic representations called into existence in the shape of dim pains to which individual experience has yet given no specific outlines.

*Representative feelings*, comprehending not only the ideas of the feelings above classed when they are called up apart from external objects, but more especially comprehending that higher class of feelings which we ordinarily distinguish as sentiments. In these—as, for example, in the sentiment of justice—sensations have no share: there is no presentative element. This feeling is compounded out of mental states that are themselves wholly, or almost wholly, representative; it involves representations of those lower emotions which are produced by the possession of property, by freedom of action, &c.; and thus is in great part even re-representative.

This classification, here but roughly indicated and capable of much further expansion, will be found in harmony with the results of detailed analysis aided by development. Whether we trace mental progression through the grades of the animal kingdom, through the grades of mankind, or through the stages of individual growth, it is obvious that the advance, alike in cognitions and feelings, is, and must be, from the presentative to the more and more remotely representative. It is undeniable that intelligence ascends from those simple perceptions in which consciousness is occupied in localizing and classifying sensations, to perceptions more and more compound, to simple reasoning, to reasoning more and more complex and abstract—more and more remote from sensation. Aud in the evolution of feelings there is a parallel series of steps. Simple sensations; sensations combined together; sensations combined with represented sensations; represented sensations organized into groups in which their separate characters are very much merged; representations of these representative groups, in which the original components have become still more vague. In both cases, the progress has necessarily been from the simple and concrete to the complex and abstract; and as with the cognitions, so with the feelings, this must be the basis of classification.

The space which we have here occupied with criticisms on Mr. Bain's work, we might have filled with exposition and eulogy, had we thought this more important. Though we have freely pointed out what we conceive to be its defects, let it not be inferred that we question its great merits. We repeat that, as a natural history of the mind, we believe it to be the best yet produced. It is a most valuable collection of carefully elaborated materials. Perhaps we cannot better express our sense of its worth than by saying that, to those who hereafter give to this branch of Psychology a thoroughly scientific organization, Mr. Bain's book will be indispensable.—*British and Foreign Medico-Chirurgical Review.*

